# Study protocol: delayed intervention randomised controlled trial within the Medical Research Council (MRC) Framework to assess the effectiveness of a new palliative care service

**DOI:** 10.1186/1472-684X-5-7

**Published:** 2006-10-02

**Authors:** Irene J Higginson, Bella Vivat, Eli Silber, Tariq Saleem, Rachel Burman, Sam Hart, Polly Edmonds

**Affiliations:** 1 Department of Palliative Care, Policy and Rehabilitation, King's College London, Weston Education Centre, Cutcombe Rd, London, SE5 9RJ, UK; 2 School of Health Science and Social Care, Brunel University, Isleworth, Middlesex, UK; 3 Department of Neurology, King's College Hospital, Denmark Hill, London SE5 9RS, UK

## Abstract

**Background:**

Palliative care has been proposed to help meet the needs of patients who suffer progressive non-cancer conditions but there have been few evaluations of service development initiatives. We report here a novel protocol for the evaluation of a new palliative care service in this context.

**Methods/Design:**

Using the MRC Framework for the Evaluation of Complex Interventions we modelled a new palliative care and neurology service for patients severely affected by Multiple Sclerosis (MS). We conducted qualitative interviews with patients, families and staff, plus a literature review to model and pilot the service. Then we designed a delayed intervention randomised controlled trial to test its effectiveness as part of phase II of the MRC framework. Inclusion criteria for the trial were patients identified by referring clinicians as having unresolved symptoms or psychological concerns. Referrers were advised to use a score of greater than 8 on the Expanded Disability Scale was a benchmark. Consenting patients newly referred to the new service were randomised to either receive the palliative care service immediately (fast-track) or after a 12-week wait (standard best practice). Face to face interviews were conducted at baseline (before intervention), and at 4–6, 10–12 (before intervention for the standard-practice group), 16–18 and 22–24 weeks with patients and their carers using standard questionnaires to assess symptoms, palliative care outcomes, function, service use and open comments. Ethics committee approval was granted separately for the qualitative phase and then for the trial.

**Discussion:**

We publish the protocol trial here, to allow methods to be reviewed in advance of publication of the results. The MRC Framework for the Evaluation of Complex Interventions was helpful in both the design of the service, methods for evaluation in convincing staff and the ethics committee to accept the trial. The research will provide valuable information on the effects of palliative care among non-cancer patients and a method to evaluate palliative care in this context.

## Background

Palliative care services have traditionally focussed on caring for patients with cancer. In the UK, 95% of patients cared for by in-patient hospice and home care services have cancer; this percentage is similar in many other countries. However, cancer accounts for only 1 in 4 deaths, and most non-cancer deaths are from conditions where patients experience a progressive, fluctuating or chronic condition [1,2]. The symptoms experienced in far advanced heart failure, respiratory failure, progressive neurological conditions, HIV/AIDS and renal failure have much in common with those of cancer, suggesting that palliative care services might have a role[3,4]. However, there is no clear model of services to follow for these conditions, and most evidence of effectiveness of palliative care treatments and services relates to patients with cancer. Thomas and McMahon proposed a model in progressive non-cancer focussed on end of life care[5,6], whereas Skilbeck and Payne suggested specialist palliative care should be viewed as a service for those with complex symptoms or problems, especially at the end of life[7]. Whatever model is developed[8,9], palliative care services in non-cancer need development and tested in robust studies.

We therefore designed a study to develop and evaluate a new palliative care service for people severely affected by multiple sclerosis (MS) (both people with MS and their families/carers), closely linked to an established MS service run by neurologists and MS nurses. We focussed on multiple sclerosis because it is a chronic disease affecting the central nervous system affecting over 2.5 million people worldwide, and is the commonest cause of neurological disability in adults under 60 years[10]. It is associated with a wide spectrum of physical symptoms, including loss of function of limbs and in many instances bladder and bowel dysfunction, pain, spasms, swallowing and communication and cognitive difficulties, many of which are as severe as among patients with cancer[11,12]. In addition, MS may have profound emotional consequences for those affected, including disbelief, devastation, loss, forced life choices, sadness and in some instances depression and family conflict[13]. It often involves complex services and treatment choices. For some the life span with MS is unaltered. However around 20% of those affected have a progressive course from the outset (primary progressive MS), and a further 35% develop a progressive course following several years of relapsing and remitting disease (secondary progressive MS)[14,15]. For these individuals treatment options to delay or prevent further disability are currently very limited[16,17]. Rehabilitation offers much in the way of improving and maintaining function, activities and support[18]. The severity of symptoms and psychological distress of the progressive course has raised questions as to whether some people would benefit from the addition of specialist palliative care. Therefore, this study aimed to develop and evaluate a new palliative care service, integrated with existing MS, nursing and rehabilitation services for people severely affected by MS.

We present the protocol of the intervention and trial because we wish to describe the intervention and study, both to ensure independence of the results and to stimulate criticism and suggestions from the journal readers. We are aware of the challenges of evaluative design of palliative care services, in particular the use of randomised controlled trials[19,20], and the need for new methods, and we believe that lessons learned from developing this protocol will add to knowledge in the field.

## Methods/Design

### Primary aim

1. To determine whether a new palliative care and neurology service for people severely affected by MS improves outcomes, including symptoms and psychosocial support.

### Secondary aims

1. To describe the nature of people who were referred to the service and their symptoms and problems

2. To determine whether there are any changes over time in symptom control, psychosocial concerns, information provision and services received

3. To identify those aspects of the service which are most useful to people with MS, their informal carers and health care professionals

4. To provide methods for future phase III evaluation of appropriate palliative care for people with MS.

### Design

**Figure 1:**
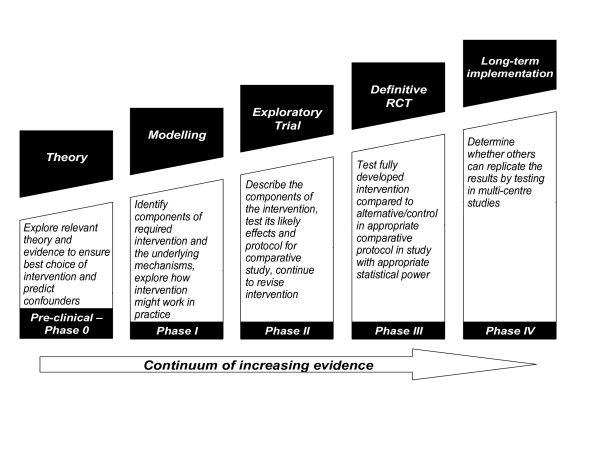
The MRC framework for the evaluation of complex interventions.

The development and evaluation of a new palliative care service for people severely affected by MS was conducted using the Medical Research Council (MRC) Framework for the Evaluation of Complex Interventions[21,22]. This approach has been used to develop and evaluate a wide variety of treatments, services and public health interventions, including prevention approaches which require behaviour change, information services, educational programmes, integrated systems of patient care and complementary therapies[22]. The MRC framework follows the approach of pre-clinical – phase IV clinical trials, see figure 1[21,22]. Although the diagram appears linear, recent work suggests that often there is iteration between the phases. In this study we aimed to complete the first three phases, from pre-clinical phases to phase II (exploratory trial).

### Project Advisory Committee

A multidisciplinary Project Advisory Committee (PAC) was established for the duration of the project. This had a lay chair, appointed by the funding body, who was a carer of a person severely affected with MS. The PAC also included representatives of the funding body, people affected by MS, clinical staff, nurses, doctors, researchers and others working in MS and related fields. The specialities of neurology, rehabilitation medicine and palliative medicine were represented on the PAC, as were local services that linked with the new palliative care service, and experts who had developed services for people more severely affected by MS in other parts of the country. The PAC met regularly and also made contact by e-mail and telephone with the project team.

### Setting

Regional care of patients with neurological diseases was organised through the Regional Neurosciences Centre for Southeast London based at King's College Hospital (KCH), where the new service was located. This Centre is the second largest regional neuroscience centre in the UK and serves an estimated population of 3.5 million people, both rural and urban and from diverse ethnic backgrounds. Patients with MS access specialist services largely through hospitals, with five out of six boroughs in the project also having established MS nurse specialists. The area has a network of palliative care services, including in-patient hospices, community services and hospital support teams, co-ordinated through the South London Palliative Care Network and other regionally based networks.

### Intervention design: theoretical and modelling (phases 0-I MRC framework)

The intervention design and modelling involved: (1) establishing a theoretical basis by reviewing (in published and unpublished literature and textbooks) the problems faced by MS patients and their families/carers and of approaches that had proved successful in related fields, such as cancer palliative care, chronic disease and rehabilitation; (2) qualitative interviews with 23 people severely affected by MS, 8 carers/family members of these individuals and a further 9 carers where patients were not interviewed (because patients were too ill to be interviewed and in one instance the patient had died); and (3) twelve focus groups and five face-to-face interviews with relevant healthcare professionals and stakeholders, including staff in primary care, care homes, neurology and rehabilitation medicine, palliative care and hospices, including a range of medical, nursing, and physiotherapy and occupational therapy staff. The interviews and focus groups used a qualitative approach. Topic guides asked about the most important concerns for people living with MS, the needs of their carers and the problems faced by staff. The results from this work led to the modelling of the service[23,24]

### Randomised controlled trial (phase II)

The benefits and difficulties of experimental and quasi-experimental trial designs were appraised (see figure 1 and table 1). In doing this we were concerned about the ethics of withholding a potentially valuable service that had been publicised to a group of patients with advanced disease and often unmet needs. After considerable debate and review of the likely effects of the service it was agreed that a delayed intervention randomised controlled trial was the best option because it provided a randomised control trial method. All patients were likely to receive the service, given their probable survival. Thus patients were randomised to either receive the intervention immediately (fast track) or to receive standard best practice alone for three months and then be offered the intervention (standard best practice). If staff and the consultant screening the referral deemed that patients had very urgent needs or were deteriorating rapidly, then immediate referral to the service was possible, and patients did not enter the trial. Reasons for immediate referral, refusal and other reasons for not entering the trial were recorded.

**Table 1:** Appraisal of design options for exploratory trial in phase II

	Pros	Cons
**Experimental designs**		
Traditional randomised controlled trial	Gold standard way to understand a difference between intervention and control	Concerns regarding recruitment, patients/staff may not be willing to take part if some patients do not get intervention, some staff had ethical concerns
Cluster randomisation	Reduce problem of disappointment of no service and contamination	Need extremely large sample and number of clusters, analysis required at level of cluster
Patient preference randomisation	Makes explicit problem of patients who have strong preference for one type of service	Difficult for patients to have a preference when they know little about service, large sample size needed, potential for staff or others to advise patients to have a particular preference
Delayed intervention randomised trial	All patients will eventually receive service, uses a gold standard methodology, it is common in this condition for patients to wait 3 months for appointments, longer survival means patients likely to actually receive service	Some staff not happy for patients to wait 3 months, effect of service must be apparent before 3 months (i.e. before control group receive intervention)
**Quasi-experimental designs**		
Geographical comparison	No problems of randomisation, potential to increase sample size by study in an area where no service	Biases involved in variations in service provision between areas
Historical controls	No problems of randomisation	Biases in data collection and potentially in sample selection
Matched controls	No problems of randomisation	Biases in patient selection, difficulty of matching
Observational study	No problems of randomisation	No comparison group, only comparison with how patients were at referral, problems of regression to the mean, interviews and inclusion in study may have effect in itself.

#### Randomisation

The randomisation was conducted independently by statistical colleagues after the baseline interview, independent of the research and clinical team, using the minimisation method[25] to give an equal balance between groups of the following: gender, age, and date of diagnosis and according to whether patients could or could not communicate. The minimisation method ensures a balanced distribution of selected potentially prognostic factors even in small trials, such as this one[25].

#### Recruitment, consent and baseline interviews

A consultant in palliative medicine (PE or IJH) not part of the service initially screened all referrals. Patients were then sent a letter giving information about the trial and the new service and inviting them to participate. Large print formats of the information were available on request. The interviewer telephoned patients several days after receipt of the letter, and arranged to meet them (usually at home) to explain more about the study and service, agree consent, ask if the nearest carer/family member could be approached, and complete the baseline interview.

#### Inclusion criteria

Patients in South East London who were living with MS and were deemed (by staff – MS nurses, neurologists, rehabilitation staff, primary care staff, social workers – and in a few instances via voluntary groups and self referrals) to have possible palliative care needs. Referrers were encouraged to identify people as severely affected by MS based on their clinical need, rather than relying on any standardised measures of disability. However, since a large Canadian population study identified that approximately 15% of people with MS have an Expanded Disability Scale Score of 8 or more (out of a possible 10)[26], this was also suggested to referrers as a benchmark for disability that would prompt consideration of referral. Examples of palliative care needs were given as uncontrolled symptoms, psychosocial concerns, advance planning and end of life issues, progressive illness or complex needs. Recruitment was aided by awareness of the service being raised by the service-modelling phase, a service launch, liaison with voluntary organisations, especially local MS Society groups, and a programme of educational events.

#### Exclusion criteria

Those patients deemed as having urgent needs (following independent review by a consultant in palliative medicine) because of rapid deterioration or severe symptoms were seen immediately by the service.

#### Standard best practice

People affected by MS within the study area received a variety of services. These were available to all those who received the new palliative care service immediately and after a delay. Amongst the services available were nurses (including nurses specialising in MS), physiotherapy, neurology and rehabilitation services. On average MS specialist nurses contacted people with MS by telephone at least once every 6 months, more if patients had greater need, and visited when appropriate. In addition, district nurses, social services  and general practitioners provided support in the community. A few patients received home physiotherapy, occupational therapy and/or attended specialist rehabilitation services or clinics. Most had seen a neurologist within the last 6 months and many were still under the care of the neurologist, although hospital attendance as an out-patient was more difficult for those with severe disability. In-patient care was available as required, particularly for the treatment of infections, although this was seldom on wards with specialist knowledge of MS or disability. In-patient rehabilitation was available to some patients, as were other specialist services, including continence advice, psychiatry and/or psychology. Most patients were entitled to some financial benefits because of their disability, which went towards their costs of care. Charities such as the MS Society provided information on available services and organised support groups. In addition, carers, usually family members and friends, supported many patients.

#### Intervention: the new service

The new service was offered in addition to the standard best practice services outlined above. It aimed to complement the existing local services and not to duplicate or replace them, providing consultation and shared care with other agencies. It comprised a part-time palliative medicine consultant (who had received training in palliative medicine and had experience in neurology), a nurse with immediate experience of working as a nurse specialist in neurology, and with some palliative care training, a psychosocial worker (also part of the existing palliative care team), and a service co-ordinator/administrator. Patients were visited by the service in their own homes, or in some instances, depending on circumstances, were seen in day units, rehabilitation centres, nursing homes or in hospital. After an initial assessment, treatment was recommended and follow-up occurred as required. The services aimed to provide a quality comprehensive palliative care assessment and suggest ways to improve management of physical, emotional, social and other problems, provide specialist welfare benefits advice and bereavement support, liaise with and act as a catalyst with local services, both primary and specialist teams, enable crisis prevention and to develop education and support for primary and secondary care. During care from the service, those patients requiring review by a neurologist were referred to ES (a consultant neurologist based at KCH) and those requiring ongoing specialist palliative care input were referred to existing local community or hospital palliative care teams.

#### Timing of follow up interviews and receipt of new service

**Figure 2:**
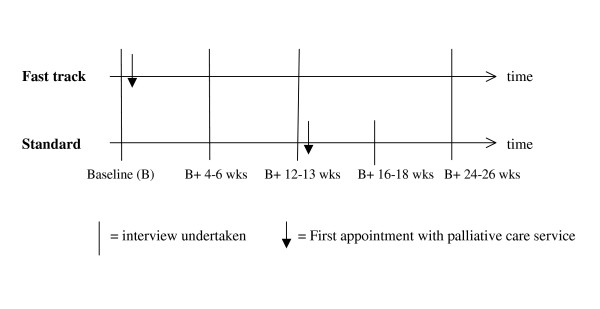
Timing of interviews and intervention for patients in both fast track and standard best practice groups.

After the baseline interview, details of those patients randomised to fast track were immediately passed to the palliative care service. Those patients randomised to standard best practice were notified and details were kept with the research team until after the third research interview at 12 weeks, when details were passed to the clinical team. Interviews were repeated for both groups at 4–6 weeks, 10–12 weeks, 16–18 weeks (the standard practice group only, to occur 4–6 weeks after receipt of the palliative care service) and 24–26 weeks. The timing and procedure is shown in figure 2.

#### The interviewers

The interviewers were all trained interviewers from either social science, nursing or medical backgrounds. All had previous experience in interviewing in palliative care. Support for interviewers, if they found the experience difficult, was offered within the department, either by other staff and, if required, using external supervision. Because of the high number of interviews needed at certain times of the study, and because of staff changes, 10 different interviewers were used at stages of the project. However, most interviews were conducted by one interviewer.

#### Trial data collection and outcome measures

**Figure 3:**
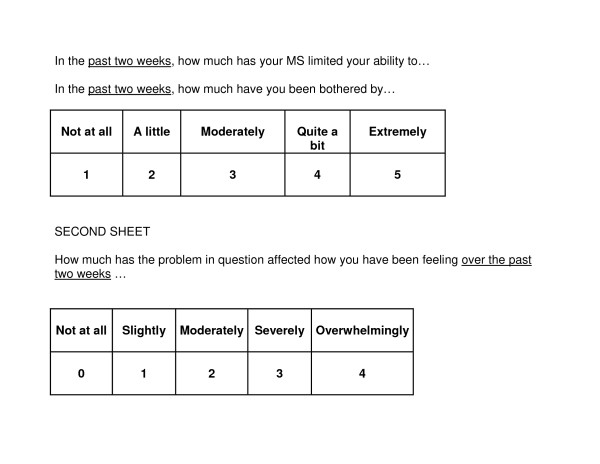
Examples of the laminated questions which patients viewed while the questionnaire was read to them.

Face to face interviews were conducted in the location most suited to the patient and/or carer, usually home, but occasionally a care or nursing home. Data collected included standardised questionnaires recording demographic, clinical data and cognitive status, and the patient's functional status (both self report and interviewer assessed[27]) (see table 2). Outcome measures were selected following a systematic literature review appraising potential questionnaires[28] and piloting of candidate measures and whole interview schedule. The primary outcome measure was palliative care symptoms as assessed by the POS-MSS, adapted to take account of symptoms relevant in palliative care and MS[29,30]. Secondary outcomes included self-reported quality of life and impact of MS[31,32], psychosocial palliative care outcomes[29,30], use of health and social services, and experience of hospital services. Not all questionnaires were used at every interview (see table 2 for full details). Carers self-completed a short separate questionnaire assessing carer burden and mastery, either whilst the patient was being interviewed, or subsequently, returning the questionnaire by post. Interviewers usually read out the questionnaire to patients. The interview process was improved by showing respondents the potential range of standard responses to some of the scales, using large print (because of visual difficulties) laminated A4 sheets (see figure 3 for an example). We timed a sample of interviews, and recorded place of interview.

**Table 2:** Questionnaires used in the trial. For a full review of measures see [28]

**Patient questionnaires**
Administered once only
• **AMTS **(Abbreviated Mental Test Score)
- 10 simple questions used to assess cognitive function
• Structured interview of basic demographic and clinical information
Administered twice (first and last interview)
• **UNDS **(United Kingdom Neurological Disability Scale) [18]
- 12 sections designed to assess disability in people with MS
• **EDSS **(Expanded Disability Status Scale) [27]
- 10-point rating scale used to identify level of MS disability

Administered at every interview
• **MSIS **(Multiple Sclerosis Impact Scale) [31, 32]
- 29 questions on a variety of MS-related symptoms on a 1–5 scale
• **POS **(Palliative Care Outcome Scale) [29, 30] + **POS-MS symptoms**
- 10 items on anxiety, patient and carer concerns, practical needs
- 18 questions specifically relating to MS symptoms on a 0–4 scale
• **Structured **health/social services/demographic **interview**
- Record of frequency and types of heath/social services received
- Assessment of hospital care if received

**Carer/family questionnaires (administered at every interview)**
• **CBurden **(Zarit Carer Burden Inventory)
- 12 questions on carer burden
• **Mastery **(Lawton caregiver mastery scale)
- 4 items on positive experiences of caring

#### Clinical staff data collection and final survey

In addition, the clinical staff recorded routine clinical data – demographic, clinical and symptom assessments – on all patients when seen by the palliative care service, including those excluded from the trial because of urgent needs or refusal. At the end of the study we independently surveyed all patients and carers who had received the service using a semi-structured questionnaire to ask about their experiences and views.

#### Ethics

The King's College Hospital Research Ethics committee gave full ethical (IRB) approval for the study. Approval was obtained separately for the different components of the study. Following piloting of the questionnaires and recruitment methods, protocol amendments were made and approved.

#### Analysis

The analysis was conducted separately for the different phases of the study.

**1**. **Qualitative data **from interviews and focus groups were analysed into themes using the constant comparative approach. Data were initially coded as 'free nodes', and these were then grouped into broader themes. This information was then used to model the service.

**2**. **The trial data **were cleaned, checked for coding errors and entered into SPSS. Initial inspection of the data checked for discrepancies in coding, and explored patterns of missing data. Using univariate and multivariate analysis we tested for factors associated with any missing data. We explored different methods of imputation of missing data, and conducted all analysis excluding missing data and also using imputations to check whether there were differences between the results using different approaches. We tested for differences between the standard practice and fast track groups at baseline.

**3**. Data were then analysed at two time points. First, data from the fast-track and standard practice groups were described and compared, plotting the severity of symptoms and problems. We tested for significant differences between fast track and standard best practice at baseline and at 4–6 weeks (interview 2) and 12 weeks (before the standard best practice patients received the service, interview 3). We also calculated response scores, computing both the difference and the ratio between baseline and interview 2 and interview 3[33]. We then compared these between our fast track and standard intervention groups. Second, we described the scores over time in the two groups, up to interview 5, contrasting the patterns and change in scores for the standard track group before and after the intervention, and for the fast track group after the intervention (up to 24 weeks)[33]. We plan to describe response scores for patients and families in subgroups, if possible, for example, according to severity of baseline problems.

Throughout we used mean (SD), median, mode and range summary statistics, independent t-tests and paired t-tests for numerical normally distributed data, and Mann Whitney U and Wilcoxon Paired Rank Test (for ordered categorical data) and Chi-Squared tests (for categorical data)[33]. Spearman (and checked with Pearson) correlations and ANOVA were calculated to test for associations between degree of missing data and patient or other characteristics.

The trial was designed to be a preliminary phase II trial, to test design feasibility, recruitment and attrition, to help model the service for the future and to determine likely effects. Therefore, a formal sample size calculation was not essential. However, we estimated that a sample of 25 patients in each group would enable us to detect clinically significant differences of greater than 1.6 on the Palliative Outcome Scale (for individual items), where items had a standard deviation of less than 2, at p < 0.05, power 80%. Based on the local patient numbers of people with an EDSS of > 8, we estimated we would identify 3–4 patients per week, and be able to recruit and follow up 2 of these. Recruitment over 1 year would therefore give us 50–52 patients, which should give us a sufficient indication whether differences between groups were emerging.

Data from the final survey and clinical activity were analysed descriptively to better understand how the new services had worked, what work the team had undertaken and what aspects recipients had found helpful, not helpful and to record their suggestions for improvement[34].

### Results from phase I and service staff recruitment

The results of phase I identified five main concerns for patients and families; loss and change, support needs (both emotional and practical), information needs (for services, aids, adaptations, benefits, and end-of-life planning), symptom control, and issues concerned with the delivery of care including co-ordination, continuity, and problems with inpatient care[35]. The focus groups and interviews with staff identified two issues similar to those of patients and a further four issues which were slightly different (see table 3)[36]. These results led us to model the service and also to hold awareness raising and educational events for staff.

**Table 3:** Issues raised by health professionals in focus groups and individual interviews when asked

Issues raised which were similar to those from patients	- continuity of care - service delivery
Issues raised which were different to those from patients/families	- resources – a concern that expanding palliative care to people affected by MS would drain their resources, or divert resources from other fields such as rehabilitation medicine - unpredictability of disease process – being unclear when a referral to palliative care would be appropriate, being unsure what to do if patients might improve or progress - mutual lack of knowledge between different specialities - need for professional training and information exchange

Initially a consultant in palliative medicine (RB) was recruited to the service. She conducted some of the staff interviews and began a consultation service for patients to further explore needs and test out methods of working in palliative care. These patients (25 in total) were not included in the trial, but were involved in piloting the questionnaires and recruitment methods. Specialist psychosocial input was obtained from the psychosocial worker on the generic hospital palliative care team. The consultant and the psychosocial worker worked part-time between the MS service and the general palliative care team. Finally, a clinical nurse specialist and an administrator working exclusively with the MS service were appointed and the trial commenced.

## Discussion

The MRC framework for the evaluation of complex interventions has been used to develop and evaluate services for patients with chronic conditions, including stroke. It has been proposed as possible in palliative care[37]. However, a search of Medline from 1966 – July 2006 found no formal reports of its use in palliative care, hospice care or terminal care. This study demonstrated how the framework might be used in modelling and evaluating a new palliative care service. There were several positive aspects to the approach. The Service Modelling phase gave valuable information on the detail of the structures and processes of the service, and in addition helped to raise awareness of the service. There was an opportunity for a wide range of disciplines, including rehabilitation medicine, neurology, palliative care, professionals allied to medicine, general practice and various branches of nursing to feed into the design of the service. We believe this helped to improve working relationships with other services. The local patient data helped to confirm local need and the precise modelling of the service. In phase II, embarking on a preliminary evaluation, was, we feel, more cost-effective than attempting a definitive stage III randomised controlled trial. The phase II nature of the trial will allow us to test the feasibility of trial methods, and the data from this trial will aid selection of design, measures and calculation of sample sizes for a larger definitive trial should this be appropriate.

Tilling et al used the MRC Framework to develop a family support organiser service and to refine outcome measures for evaluation[38]. After development they progressed directly to a phase III randomised controlled trial, and the preclinical and second phases were very limited. Robinson et al sought to complete the early phases of the MRC Framework to facilitate coping skills in new carers of stroke patients[39]. In the preclinical (theoretical) phase, a theoretically based framework for a small group course for carers of people with stroke was developed. The intervention was grounded in a cognitive behavioural model and included carers' needs identified from a literature review. Phase I (modelling phase) comprised a qualitative study involving one-to-one semi-structured interviews with a purposive sample of informal carers of people with stroke. Following this, the intervention was modified. In phase II (exploratory phase), the modified intervention was delivered by a clinical psychologist and stroke nurse practitioner to five carers. The course was further refined and delivered to seven new carers who subsequently completed a satisfaction questionnaire. As in our study the MRC framework provided a useful methodology for the development of a complex intervention.

A criticism of our study is the limited theoretical development at the start of pre-clinical modelling. We did model the service on evidence of other services that helped patients and families, and on some theoretical constructs, but this could have been more formally done. However, the modelling in phase I was more extensive than that of Tilling et al[38] or Robinson et al[39], compensating for this deficit. Furthermore, our preliminary evaluation (phase II) was far more extensive than either of these studies. Robinson et al interviewed only 12 carers, and Tilling et al virtually omitted the phase. However, given the difficulty of trials in palliative care [40, 41, 42], and the number of trials that experience serious problems with recruitment, attrition and contamination, we feel that a detailed phase II study was warranted. In the development of drugs and other therapies, phase I and phase II studies are often given considerable time and attention, and findings are often published in very high impact factor journals. It is time, perhaps that such attention is given to palliative service developments at phase I and II. By introducing a randomised trial at phase II we will be able to test the trial method as well as the intervention.

The use of a delayed intervention randomised trial in palliative care is also novel. We are aware of no other study in palliative care that has used this design. Often this approach is not realistic; palliative care patients usually die so quickly that those in the delayed intervention group will not receive the intervention. However, the design is used in rehabilitation studies, and this group of patients was accustomed to wait for longer periods for outpatient appointments etc. Therefore, this design was deemed as appropriate and was acceptable to people with MS, carers and professionals. We suggest that as palliative care moves forward into non-cancer conditions, where survival is longer and less easy to predict, this design would be of value.

## Competing interests

The author(s) declare that they have no competing interests.

## Authors' contributions

IJH co-conceived the study and design, co-applied for funding, oversaw all study conduct and developed the analysis plan. PE co-conceived the study and design, led the application for funding and oversaw all conduct of the study. BV was appointed as research fellow on the project, conducted and analysed interviews for phase I, drafted ethics applications and assisted in the detailed design of phase II. ES co-applied for funding and contributed to the design and study conduct. TS was appointed interim research fellow on the project after BV left, and helped to refine interview techniques in phase II. RB was appointed as consultant to the service, conducted and analysed interviews for phase I, collected clinical data and oversaw the clinical service activity. SH was appointed as research associate on the project, entered and analysed data in phase II, and contributed to the design of and ethics submission for the post interview survey. SH, TS, BV, IJH and PE all conducted interviews in phase II. IJH led the drafting of this paper (working closely with PE, RB and SH). All other authors saw or contributed to or commented on the final draft. IJH acts as guarantor.

## Pre-publication history

The pre-publication history for this paper can be accessed here:


